# Complete Genome Sequences of *Campylobacter jejuni* Strains OD267 and WP2202 Isolated from Retail Chicken Livers and Gizzards Reveal the Presence of Novel 116-Kilobase and 119-Kilobase Megaplasmids with Type VI Secretion Systems

**DOI:** 10.1128/genomeA.01060-16

**Published:** 2016-09-29

**Authors:** Daya Marasini, Mohamed K. Fakhr

**Affiliations:** Department of Biological Science, The University of Tulsa, Tulsa, Oklahoma, USA

## Abstract

Genome sequences of *Campylobacter jejuni* strains OD267 and WP2202, isolated from chicken livers and gizzards, showed the presence of novel 116-kb and 119-kb megaplasmids, respectively. The two megaplasmids carry a type VI secretion system and tetracycline resistance genes. These are the largest sequenced *Campylobacter* plasmids to date.

## GENOME ANNOUNCEMENT

*Campylobacter* spp. are the third most common bacterial pathogens contributing to foodborne illnesses in the United States with an estimated 845,000 annual cases (1). Our laboratory has been involved in several studies that have resulted in the isolation of a large number of *C. jejuni* and *C. coli* strains from various retail meats (2–5). Large plasmids were detected on several of these strains (6, 7). A type VI secretion system was recently characterized in *C. jejuni* and is believed to play important roles in virulence and cytotoxicity (8–10). Type VI secretion systems are mainly present in the chromosomes of various bacteria, but very limited studies have recently reported its presence on plasmids (11, 12). We announce here the complete genome sequences of two *C. jejuni* strains isolated from retail chicken livers and gizzards, respectively, and harboring novel 116-kb and 119-kb megaplasmids with type VI secretion systems. To our knowledge, these are the largest sequenced *Campylobacter* plasmids to date.

Total genomic DNA isolation was performed using the DNeasy Blood and Tissue kit (Qiagen, Valencia, CA, USA). Plasmids were also isolated separately for verification using the Qiagen Plasmid Midi kit. Genomic libraries were prepared using the Nextera XT sample preparation kit (Illumina Inc., San Diego, CA, USA). Libraries were subjected to next-generation sequencing using an Illumina MiSeq desktop sequencer and the Illumina V2 reagent kit with 2 × 150 cycles (Illumina Inc., San Diego, CA) and >100× coverage. Assembly was performed using the CLC Genomics Workbench version 7.5.1 and the Microbial Genome Finishing Module version 1.4 (Qiagen). Annotation was done using RAST and the NCBI Genome Annotation Pipeline.

The complete genome sequence of *C. jejuni* isolate OD267 showed the presence of a circular chromosome of 1,672,837 bp in size and two plasmids: pCJDM67 L (116,833 bp) and pCJDM67 S (36,603 bp). The chromosome contained 1,786 genes, 64 pseudogenes, and 54 RNAs and had a G+C content of 30.5%. The megaplasmid pCJDM67 L contained 108 genes and nine pseudogenes. The smaller plasmid pCJDM67 S is a pVir homologue and contains 46 genes and one pseudogene. Genomic sequencing of *C. jejuni* isolate WP2202 contained a chromosome of 1,681,907 bp in size and a megaplasmid pCJDM202 (119,543 bp). The chromosome contained 1,781 genes, 42 pseudogenes, and 56 RNAs and had a G+C content of 30.5%. The megaplasmid pCJDM202 L contained 116 genes and 11 pseudogenes. The two megaplasmids sequenced in this study harbored genes encoding tetracycline resistance, lysozyme, the cag pathogenicity island protein, and all the 14 core genes of the type VI secretion system. The presence of type VI secretion systems on these mega *Campylobacter* plasmids is very interesting and suggests a role for these plasmids in the pathogenicity of this important foodborne pathogen.

### Accession number(s).

The GenBank accession numbers of the chromosomes and plasmids of the *C. jejuni* isolates sequenced in this study are as follows: OD267 (CP014744 [chromosome], CP014745 [plasmid pCJDM67 L], CP014746 [plasmid pCJDM67 S]), and WP2202 (CP014742 [chromosome] and CP014743 [plasmid pCJDM202]).
